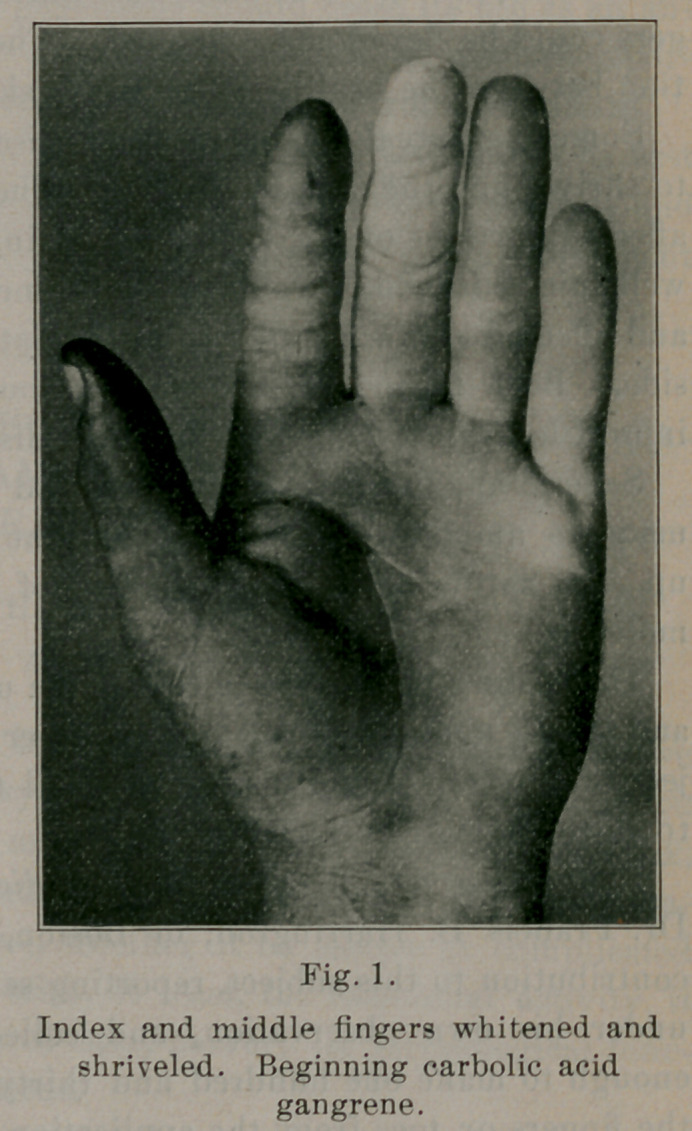# Carbolic Acid Gangrene

**Published:** 1901-06

**Authors:** W. H. Hudson

**Affiliations:** LaFayette, Ala.


### CARBOLIC ACID GANGRENE.

### GANGRENE OF THE FINGERS AND TOES PRODUCED BY THE USUAL DILUTE SOLUTION OF CARBOLIC ACID.

BY W. H. HUDSON, M.D.,
LAFAYETTE, ALA.

That the usual dilute solution of carbolic acid, when applied to the fingers and toes for any length of time, may cause gangrene and loss of these members seems to be a fact very little known among physicians, much less by the people at large.

It has been only a few years since I read an article—I do not remember the author's name—advising, as a routine practice, the treatment of lacerated and contused fingers by the constant application of a moist dressing of carbolic acid solution.

These cases of gangrene are not produced by the application of the pure carbolic acid, as would at first thought be expected, but by the watery solution of 5 per cent. strength, or less. The cases reported are very similar in their history: for some injury, usually very slight, a bandage is applied to the finger, wet in a weak solution of carbolic acid, with instruction, generally, to keep the dressing wet from a few hours to a day or two with the carbolic acid solution. The pressure of the bandage, in these cases, does not play an important part, although it may help, to a certain extent, to bring about the gangrene; after some hours the sensibilities of the finger are somewhat interfered with. Then the skin whitens and shrivels, and from this time on these conditions increase, until there is a distinct sign of demarkation between the healthy skin and the affected portion. A little later the skin darkens, the nail comes off and the finger has a leathery appearance and feel. The finger may still be extended or flexed, but the sensibilities are entirely gone, and the line of demarkation is distinctive. The affected portion of the member continues to shrivel, and unless amputation has been done earlier will come off of itself, the bone remaining up to the first joint within the gangrenous portion.

I know of no subject more important at the present time than this, and, while the loss of fingers and toes must be comparatively infrequent, the accident, nevertheless, must occur now and then; and, I think, has, in the majority of cases, been attributed to the pressure of the bandage, or to the traumatism, for which the finger is treated.

I have spoken to many physicians of this carbolic acid gangrene, and rarely one knew that such a thing was at all probable. The people look upon the carbolic acid solution as a safe household remedy for bruises, cuts, etc., and the physicians and the people should, by all means, be fully aware of this danger from the use of carbolic acid, even in the mildest solution.

Fig. 1.

Index and middle fingers whitened and shriveled. Beginning carbolic acid gangrene.

The case that came under my observation is as follows: A young white man twenty-two or -three years old consulted me in the summer of 1900. He was a railroad hand, and eight or ten days previous to the time of consulting me had received a slight injury to the first and middle fingers of his left hand while following his duties.

The section boss put a bandage on the two fingers and wet it with a solution of carbolic acid from a bottle which he kept for the purpose in treating such slight injuries. Two or three times during the night, and next day, the bandage was rewet with the carbolic acid solution.

After a few hours the fingers became numb and appeared whitened. This condition increased to that extent that the young man became alarmed and consulted several physicians.

Eight or ten days after the injury he consulted me. At that time a photograph, from which Fig. No. 1 was taken, was made. The index and middle fingers were entirely anesthetic to every form of sensation. The skin was white and leathery, but the fingers could be flexed and extended. There was distinct demarkation between the healthy and affected skin.

Some ineffectual treatment was advised. The fingers continued to shrivel and the skin to darken. The finger-nails came off, the affected portions of the fingers presenting a very good picture of a well-injected hand after it had dried for some time. The healthy and affected skin separated and granulations formed on the healthy side. Both fingers came off about four weeks after the primary injury, leaving the bones to the next distal joint sticking out.

Several appointments, by myself and other physicians, had been made to amputate the fingers, but the man always failed to turn up. So nature had an opportunity of following her course unmolested.

The history of the above case is the usual history of such cases, and it will not avail much to go further into the study of this subject than to report the case, and to call the attention of physicians to this danger from carbolic acid.

In the *American Journal of the Medical Sciences* for July, 1900, Dr. Francis B. Harrington, of Boston, makes a very instructive contribution to this subject, reporting several cases which had been under his own observation, and collecting from the literature enough to make one hundred and thirty-two cases of gangrene of the fingers or toes from the application of dilute solutions of carbolic acid.

The opinion has been expressed that the gangrene was due to the action of carbolic acid upon the trophic and vascular nerves of the part, but recent observations go to show that the gangrene is produced by its direct action upon all the tissues of the part, inducing stasis and thrombosis, and thereby interfering entirely with nutrition.

That gangrene is not produced in other portions of the body is due to the fact that the nutrition is kept up by the underlying blood-vessels, which the carbolic acid solution is not able to destroy.

Again, I will insist that carbolic acid, in the hands of the laity, is a poisonous drug, and should not be used by them for any purpose whatsoever, and that physicians should cease its use as an antiseptic in any form on the fingers and toes, and on any other portion of the body, except the flat surfaces of the trunk and limbs.

Indeed, I may say that the use of carbolic acid would be best restricted to the use of the pure acid, at points of very active and violent infection, and then to be neutralized by the subsequent application of alcohol.

## Figures and Tables

**Fig. 1. f1:**